# Data on the circulating levels of endothelial microparticles are elevated in patients with bicuspid aortic valve and are related to aortic dilation

**DOI:** 10.1016/j.dib.2016.06.026

**Published:** 2016-06-23

**Authors:** Josep M. Alegret, Neus Martínez-Micaelo, Gerard Aragonès, Raúl Beltrán-Debón

**Affiliations:** Grup de Recerca Cardiovascular, Hospital Universitari de Sant Joan, IISPV, Universitat Rovira i Virgili, Reus, Spain

## Abstract

The data included here support the research article “Circulating endothelial microparticles are elevated in bicuspid aortic valve (BAV) disease and related to aortic dilation” (Alegret et al., 2016 [1]) where circulating levels of platelet endothelial cell adhesion molecule (PECAM^+^) endothelial microparticles (EMPs) were identified as a biological variable related to aortic dilation in patients with BAV disease. The data presented in this article are composed by four tables and one figure containing the clinical and echocardiographic characteristics of the patients (Alegret et al., 2016 [1]) included in this study, and summarize the results of multivariate linear analyses. Furthermore, is also included a figure showing a representative flow cytometry dot plots and histograms used in PECAM^+^ EMPs quantification is also included.

**Specifications Table**

**Table:** 

Subject area	*Biology*
More specific subject area	*Cardiology*
Type of data	*Table*, *figure*
How data was acquired	*Flow cytometer*; *EPICS*-*XL* (*Beckman Coulter*)
Data format	*Analyzed*
Experimental factors	*Endothelial microparticles were isolated from platelet*-*poor plasma from healthy controls and patients with bicuspid aortic valve* (*BAV*) *with or without aortic dilation.*
Experimental features	Clinical and echocardiographic characteristics were determined for *healthy controls and patients with bicuspid aortic valve* (*BAV*) *with or without aortic dilation.*
Data source location	*Spain*
Data accessibility	*Data are within this article*

## Value of the data

• The data provide a novel biological variable related to aortic dilation in the setting of BAV disease.
• From the quantification of the circulating PECAM^+^ EMP levels new biomarkers of aortic dilation and endothelial dysfunction in BAV disease can emerge.
• The data provide a link between BAV disease and endothelial aortic root damage and aortic dilation.

## 1 Data

The article includes the clinical and echocardiographic characteristics of the patients included in the study [1], and the summaries of the results of multivariate linear analyses. Furthermore, is also included a figure showing a representative flow cytometry dot plots and histograms used in PECAM^+^ EMPs quantification.

## 2 Experimental design, materials and methods

### 2.1 Study population

This article includes a cohort of patients with BAV and patients with aortic dilation who were prospectively included and followed-up in our facilities. There were 185 patients with BAV and 125 patients with aortic dilation with tricuspid aortic valve (TAV). The participants were prospectively entered into a specific database and provided a blood sample upon enrollment and written acceptance.

The experimental design included four evaluations to determine data related to the EMP circulating levels and BAV disease and are completely described in the research article “Circulating endothelial microparticles are elevated in bicuspid aortic valve disease and related to aortic dilation” [1].

### 2.2 Blood sampling and EMP identification

EDTA(K_3_)-blood samples were collected following overnight fasting and were processed within 90 min of collection. Samples were centrifuged at 1500*g* for 15 min to obtain plasma, which was further centrifuged at 4000*g* for 10 min to obtain platelet-poor plasma. The samples were stored at −80 °C until needed in our biological samples bank (Biobanc IISPV - HUSJR).

The concentration of circulating EMPs was determined on an EPICS-XL (Beckman Coulter) flow cytometer at low rate setting and 30 s stop time. The Nano Fluorescent Particle Size Standard Kit (Spherotech) was used for instrument standardization and the Flow-Count fluorospheres (Beckman Coulter) were added as an internal calibrator to calculate microparticles amount.

Plasma EMPs were labeled by incubating 50 μl of platelet-poor plasma with the corresponding antibody: anti-CD31-PE (Beckman Coulter), anti-CD42b-FITC (Beckman Coulter) and anti-CD45-PE (Beckman Coulter) at room temperature in the dark for 20 min as previously described [2]; 500 μl of PBS was subsequently added, and the EMP levels were determined quantified as previously described [2], [3], [4]. Anti-CD62E-PC5 (Abcam) was used in order to determine the EMPs released from activated endothelial cells (E-selectin^+^). Microparticles that expressed phosphatidylserine were labeled using the Annexin V apoptosis kit (Abcam) in the presence of CaCl_2_ according to the manufacturer׳s recommendations.

Once microparticles were labeled appropriately, events <1 μm were identified via forward scatter and side scatter intensity dot representations using gated microparticles and plotted on either one- or two-color fluorescence histograms (Supplementary Fig. 1). EMPs were defined as particles >0.1 and <1 μm in size and the endothelial origin was identified based on its affinity to CD31^+^CD42b^−^ (PECAM^+^), specific cell surface antigens. In order to determine the possible contamination of leukocyte-derived microparticles the circulating levels of CD31^+^CD45^+^ was determined in all samples obtaining levels <4.5% of CD31^+^ microparticles co-expressing CD45^+^, this results are in accordance to that observed previously by other authors [5], [6]. EMP levels were measured by trained technicians in a blinded fashion to clinical statutes of the patients as well as to the results.

### 2.3 Statistical analysis

Because of the right-skewed distribution of the values, the EMP plasma levels underwent a natural logarithmic transformation and were expressed as log-transformed counts *per* μl (log EMPs/μl). The quantitative variables are represented as means±SDs. The means of two groups were compared using Student׳s *t*-test. Chi-squared tests, or Fisher exact test when appropriate, were used to compare the frequencies of the categorical variables. Backward linear regression models were used to identify independent predictors of the circulating PECAM^+^ EMP levels. *P* values<0.05 were considered significant. The statistical analysis was performed using SPSS software, version 21.0 (IBM, Chicago, IL, USA).

## Footnotes

Appendix A Supplementary data associated with this article can be found in the online version at http://dx.doi.org/10.1016/j.dib.2016.06.026.
