# Supplementary material for: Data on the circulating levels of endothelial microparticles are elevated in patients with bicuspid aortic valve and are related to aortic dilation
Source: Data Brief. 2016 Jun 23;8:666–9. doi: 10.1016/j.dib.2016.06.026 (PMC4943086; doi:10.1016/j.dib.2016.06.026)
Supplement: Supplementary file 1 — Supplementary material [file mmc1.doc]

## Conflicts of interest: none
