# Supplementary material for: Data on the circulating levels of endothelial microparticles are elevated in patients with bicuspid aortic valve and are related to aortic dilation
Source: Data Brief. 2016 Jun 23;8:666–9. doi: 10.1016/j.dib.2016.06.026 (PMC4943086; doi:10.1016/j.dib.2016.06.026)
Supplement: Supplementary Figure 2 — Supplementary material [file mmc2.doc]

**Table 1.** A summary of the results of the multivariate linear analysis of the PECAM^+^ EMP levels of the control subjects and the BAV patients (first evaluation).

|  | **PECAM^+^ EMP levels (log EMPs/uL)** | |
| --- | --- | --- |
|  | β | p |
| Age (x year) | 0.048 | 0.662 |
| Sex | -0.114 | 0.294 |
| Hypertension | 0.097 | 0.388 |
| Aortic stenosis (mean gradient ≥20 mm Hg) | -0.082 | 0.464 |
| Valve morphology (TAV/BAV) | 0.380** | 0.001 |

** Significant values (p<0.01); PECAM^+^, platelet endothelial cell adhesion molecule; EMPs, endothelial microparticles; BAV, bicuspid aortic valve; TAV, tricuspid aortic valve

**Table 2.** The clinical and echocardiographic characteristics of the patients with aortic dilation and different aortic valve morphology (third evaluation).

|  | **TAV_dil_** | **BAV_dil_** | **p** |
| --- | --- | --- | --- |
| Age (years) | 64 ± 3 | 49 ± 2** | <0.001 |
| Male gender | 14 (70.0%) | 45 (65.2%) | 0.690 |
| Weight (kg) | 76.16 ± 3.1 | 70.74 ± 1.7 | 0.137 |
| Body mass index (kg/m^2^) | 22.36 ± 1.0 | 25.62 ± 0.5 | 0.127 |
| Body surface area (m^2^) | 1.84 ± 0.1 | 1.78 ± 0.1 | 0.218 |
| Hypertension | 13 (65.0%) | 23 (33.3%)* | 0.011 |
| Hypercholesterolemia | 3 (15.0%) | 8 (11.6%) | 0.684 |
| Smoker | 4 (20.0%) | 17 (24.6%) | 0.851 |
| Treatment:  Statins | 5 (25.0%) | 14 (20.3%) | 0.651 |
| ACE/ARAII | 8 (40.0%) | 14 (20.3%) | 0.072 |
| Aortic stenosis (mean gradient ≥20 mm Hg) | 1 (5.0%) | 24 (34.8%)** | 0.009 |
| Aortic regurgitation (≥II) | 6 (35.3%) | 34 (51.5%) | 0.233 |
| Indexed aortic root diameter (mm/m^2^) | 22.36 ± 0.7 | 22.39 ± 058 | 0.969 |
| Indexed ascending aorta diameter (mm/m^2^) | 24.25 ± 1.1 | 24.40 ± 0.6 | 0.903 |
| Left ventricle diastolic diameter (mm) | 51.11 ± 1.1 | 52.61 ± 0.8 | 0.369 |
| Left ventricle systolic diameter (mm) | 31.81 ± 1.4 | 32.87 ± 0.8 | 0.533 |
| Left ventricular ejection fraction (%) | 70.94 ± 1.1 | 69.30 ± 1.2 | 0.530 |
| Mean transvalvular aortic gradient (mm Hg) | 6.58 ± 2.1 | 20.03 ± 2.1** | <0.001 |

*Significant values (p<0.05); ** Significant values (p<0.01); BAV, bicuspid aortic valve; TAV, tricuspid aortic valve

**Table 3.** A summary of the results of the multivariate linear analysis of PECAM^+^ EMP levels of the patients with TAV_dil_ and BAV_dil_.

|  | **EMP levels (log EMPs/ul)** | |
| --- | --- | --- |
|  | β | p |
| Age (x year) | -0.070 | 0.564 |
| Hypertension | 0.158 | 0.172 |
| Body surface area (m^2^) | 0.042 | 0.713 |
| Aortic stenosis (mean gradient ≥20 mm Hg) | -0.058 | 0.618 |
| Aortic regurgitation | 0.095 | 0.408 |
| Valve morphology (BAV) | -0.280* | 0.016 |
| Indexed aortic root diameter (mm/m^2^) | 0.105 | 0.363 |

* Significant values (p<0.05); EMPs, endothelial microparticles; BAV, bicuspid aortic valve; TAV, tricuspid aortic valve

**Table 4.** The clinical and echocardiographic characteristics of the patients with BAV who underwent either aortic surgery or aortic valve surgery (fourth evaluation).

| **BAV patients underwent AVS** | |
| --- | --- |
| Age (years) | 54 ± 4 |
| Male gender | 6 (60%) |
| Weight (kg) | 69.90 ± 3.2 |
| Body surface area (m^2^) | 1.75 ± 0.1 |
| Hypertension | 9 (90%) |
| Hypercholesterolemia | 9 (90%) |
| Smoker | 3 (30%) |
| Aortic stenosis (mean gradient ≥20 mm Hg) | 5 (50%) |
| Aortic regurgitation (≥II) | 4 (40%) |
| Indexed aortic root diameter (mm/m^2^) | 23.50 ± 1.7 |
| Indexed ascending aorta diameter (mm/m^2^) | 26.77 ± 2.8 |
| Left ventricle diastolic diameter (mm) | 54.44 ± 2.8 |
| Left ventricle systolic diameter (mm) | 34.56 ± 2.2 |
| Left ventricular ejection fraction (%) | 65.33 ± 4.7 |
| Mean transvalvular aortic gradient (mm Hg) | 37.22 ± 8.0 |
| Type of surgery |  |
| Aortic valve replacement | 4 (40%) |
| Ascending aortic graft | 2 (20%) |
| Aortic valve replacement + Ascending aortic graft | 4 (40%) |

BAV, bicuspid aortic valve; AVS, aortic valve surgery

**Figure 1.** Representative flow cytometry dot plots and histograms of circulating PECAM^+^ EMPs for a healthy TAV control (A, B and C) and a BAV patient (D, E and F). A and D) Size-selected events (range size of 0.1 to 1.0 μm) are plotted in accordance to their fluorescence for CD31-PE and CD42b-FITC binding on a two-fluorescence plot. Representative histograms showing the CD31-PE (B, E) and CD42b-FITC (C, F) expression on endothelial microparticles for a TAV.
